# A Proving of Lac Vaccinum Defloratum

**Published:** 1884-03

**Authors:** E. W. Berridge

**Affiliations:** London


### PROVING OF LAC VACCINUM DEFLORATUM.

E. W. BERRIDGE, M. D., LONDON.

Mrs. D. H. H. W., æt. twenty-eight, twice took three cm. (Swan) with the same result each time; the first time she took two doses; the second time number of doses not recorded. It caused photophobia for sunlight in both eyes; eyes red, lachrymative; left eye more sore than right.

